# Analysis of cranial type characteristics in term infants: a multi-center study

**DOI:** 10.1186/s12887-020-02374-5

**Published:** 2021-01-19

**Authors:** Wang Yang, Bin Hu, Jianping Chen, Wenzhi Shen, Chengju Wang, Qin Chang, Wenzao Li, Fuxiang Qu, Qiuming Pan, Yuping Zhang

**Affiliations:** 1 Department of Pediatrics, the Second Affiliate Hospital of Army Medical University, No. 83 Xinqiao Street, 400037 Chongqing, China; 2 Department of Child Health Care, Yongchuan Maternal and Child Health Care Hospital of Chongqing, 402160 Chongqing, China; 3 Department of Child Health Care, Wanzhou Maternal and Child Health Care Hospital of Chongqing, 404000 Chongqing, China

**Keywords:** positional head deformity, term infants, plagiocephaly, brachycephaly, dolichocephaly

## Abstract

**Background:**

Positional head deformity (PHD) is defined as a change in the shape of an infant’s skull due to an external force. In certain cases, it can lead to cosmetic deformities or even neurological issues due to its impact on the developing nervous system. Therefore, we conducted this study to investigate the incidence and characteristics of PHD in term infants in China and preliminarily establish a localized diagnostic reference standard.

**Methods:**

Overall, 4456 term infants from three medical institutions in Chongqing were and divided and analyzed according to their age. Cranial vault asymmetry (CVA) and cephalic index (CI) were calculated in all infants. The current international diagnostic criteria were used to understand PHD incidence and analyze the CVA and CI distribution.

**Results:**

According to the current international standards, the total detection rate of PHD in Chongqing’s term infants was 81.5%, with brachycephaly alone being the most frequent (39.4%), followed by brachycephaly with plagiocephaly (34.8%) and plagiocephaly alone (6.2%). The detection rates of dolichocephaly were low: alone, 0.9% and combined with plagiocephaly, 0.2%. According to age, plagiocephaly (44.5%) and brachycephaly (82.0%) were the most frequent in the 2-3-month group. The 75th/90th/97th and 3rd/10th/25th/75th/90th/97th percentiles of CVA and CIs were 0.4/0.7/1.0 and 76.4/78.8/82.3/91.1/94.6/99.2%, respectively.

**Conclusions:**

According to the current international standards, the PHD detection rate among term infants in Chongqing was high. Therefore, a new diagnostic standard for Chinese infants was proposed where CVA ≥ 0.4 cm indicates plagiocephaly, CI ≥ 91% indicates brachycephaly, and CI ≤ 82% indicates dolichocephaly.

## Background

Positional head deformity (PHD) refers to changes in the shape of an infant’s skull in the front, back, or sides due to an external force. PHD can lead to cranial or facial unsightliness, resulting in the child developing an inferiority complex and even being bullied by other children. Serious PHD may also be combined with impaired nervous system development and cognitive function, resulting in intellectual disabilities, learning difficulties, and/or language disorders [1, 2]. PHD is a common problem in the first six months of life and is present in 20–46% of live births [3]. The earlier the discovery of PHD, the better is the effect of the intervention and the lower is its cost [4]. After six months of age, the growth rate of the infant’s skull decreases, while the hardness of the skull and the free movement of the head increases; therefore, PHD generally does not continue to progress; however, treatment difficulty also increases compared to that before six months of age [5]. Therefore, the key period for prevention and management of PHD is up to six months of age.

PHD can be divided into three types, according to the head shape: (1) plagiocephaly, wherein because of uneven stress on both sides of the skull, one side of the skull is compressed and tilted, resulting in an increase in the difference between the skull’s diagonals; (2) brachycephaly, also known as flat head, refers to the flat shape of the skull, with an increased ratio of the head width to length; and (3) dolichocephaly, wherein the diameters of the front and back of the skull are significantly larger than the left and right diameters, resulting in a long and narrow head shape [6].

The criteria for diagnosis of PHD and degree of its severity are determined using quantitative indexes. The two main indexes for PHD diagnosis are the cranial vault asymmetry (CVA) and cephalic index (CI). Previous researchers have stated that the diagnostic standards of plagiocephaly, brachycephaly, and dolichocephaly were CVA ≥ 0.3%, CI ≥ 82%, and CI ≤ 76%, respectively [1, 7, 8]. Nowadays, these are the most widely used diagnostic standards for determining PHD type and degree globally. However, owing to large differences in the basic infant cranial types among different regions and races, it is inappropriate to diagnose infant PHD using only these standards [9]. In China, because of the impact of socioeconomic problems and insufficient knowledge regarding PHD, people lack awareness about its harmful effects. Furthermore, there have been only a few relevant clinical studies, and there is no established diagnostic standard suitable for PHD cases among Chinese infants, which means diagnosis and prevention does not occur in the most effective treatment period [10].

Therefore, we collected the cranial type measurements of term infants hospitalized in three medical institutions in Chongqing to determine the PHD incidence and analyze the cranial type characteristics, hoping to facilitate early diagnosis of and intervention for PHD in Chinese infants.

## Methods

### Research participants

**Fig. 1:**
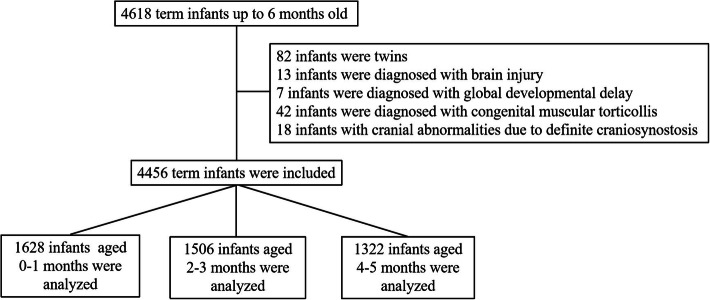
Flow diagram of the study

In this study, we enrolled 4618 term infants aged up to 6 months old who visited the outpatient departments of the primary care clinic of Xinqiao Hospital, Army Medical University and the Maternal and Child Health Care Hospitals of Wanzhou and Yongchuan in Chongqing from September 1, 2017, to August 31, 2019. Data were collected once for each infant. After screening according to the selection criteria, 4456 term infants were finally included. Among the 162 excluded infants, 82 were twins while 13, 7, 42, and 18 were diagnosed with brain injury, global developmental delay, congenital muscular torticollis, and cranial abnormalities due to definite craniosynostosis, respectively (Fig. 1). The inclusion criteria were: (1) gestational age at birth, 37–42 weeks; (2) single birth; and (3) appropriate for gestational age. We excluded infants with: (1) brain injury, dysplasia, or global developmental delay within 6 months, (2) congenital muscular torticollis, and (3) cranial abnormalities due to definite craniosynostosis.

The study was approved by the ethics committee of the Second Affiliated Hospital of the Army Medical University (Ethics approval number: 2016-研第024–01). Informed written and verbal consent was obtained from the infants’ parents or guardians. The trial was performed in accordance with the approved guidelines and regulations of the participating institutions.

### Measurement

The manual measurement method of Wilbrand et al.’s standardization scheme was adopted [11]. The measurement tool used was the KWJ124 bending foot gauge (size 260 × 260 + 36 mm); the measurement range was 0-300 mm, and the executive production standard was GB5704.3-85.

Survey personnel were intensively trained before the test. According to the reliability test, the measurement difference among survey personnel was less than 5%; each parameter was measured thrice per patient, and the mean value was analyzed.

The examiner held the infant’s head in a centered position while the infant faced the examiner. The standard measurement scheme required the following: (1) the transcranial oblique diameter, which was the distance from the middle point of the temporal ridge of the frontal bone to the inner edge of the contralateral herringbone suture, while the long and short diameters were diagonal A (DA) and diagonal B (DB), respectively; (2) the head length, which was the distance from the glabella to the farthest point (opisthocranion); and (3) the head width, which was the distance between two points 1 cm higher than the attachment point of both ears. All measuring lines were parallel to the Frankfurt line [12].

Using these values, the following were calculated: CVA, which was the difference of the oblique diameter on both sides of the head (CVA = DA-DB) and CI, which was the ratio of the maximum transverse diameter of the cranial to the maximum fore-and-aft diameter (CI = cranial width/cranial length × 100%).

### Diagnostic criteria

The diagnostic criteria were based on the *Handbook of Physical Measurements* and current international standard for PHD diagnosis [1, 7, 8, 13, 14] (Table 1).

**Table 1:** Diagnostic criteria of the type and severity of positional head deformity

	Plagiocephaly (CVA)	Brachycephaly (CI)	Dolichocephaly (CI)
Mild	3–10 mm	82–90%	74–76%
Moderate	10–12 mm	90–100%	70–74%
Severe	> 12 mm	> 100%	< 70%

*CVA* Cranial vault asymmetry, *CI* Cephalic index

### Statistical analysis

Statistical analysis was performed using IBM SPSS Statistics 22.0 for Windows (IBM Corp., Armonk, NY, USA). Measurement data are represented as mean ± standard deviation. The mean differences among the groups were analyzed using a one-way analysis of variance, and the comparisons of count data between the groups were tested using a chi-square test. *P* < 0.05 was considered statistically significant.

## Results

### Patient characteristics

A total of 4456 term infants were enrolled (0–1 months, 1628; 2–3 months, 1506; 4–5 months, 1322). The general characteristics of each group are shown in Table 2. There were no significant differences in average gestational age, birth weight, sex, and delivery type.

**Table 2:** General characteristics of term infants

	0–1 months	2–3 months	4–5 months	Statistical value
*n*	1628	1506	1322	
Gestational age (*x ± s*, w)	39.1 ± 2.2	38.9 ± 1.7	39.2 ± 0.9	*F* = 2.926, *P* = 0.4152
Birth weight (*x ± s*, kg)	3.22 ± 0.46	3.19 ± 0.71	3.22 ± 0.38	*F* = 1.876, *P* = 0.2659
Sex (Male/female, *n)*	801/827	724/782	623/699	*χ*^*2*^ = 2.240, *P* = 0.617
Delivery type (natural birth/cesarean section, *n)*	873/755	776/730	701/624	*χ*^*2*^ = 1.983, *P* = 0.752

### Cranial patterns according to the current international general standard

**Fig. 2:**
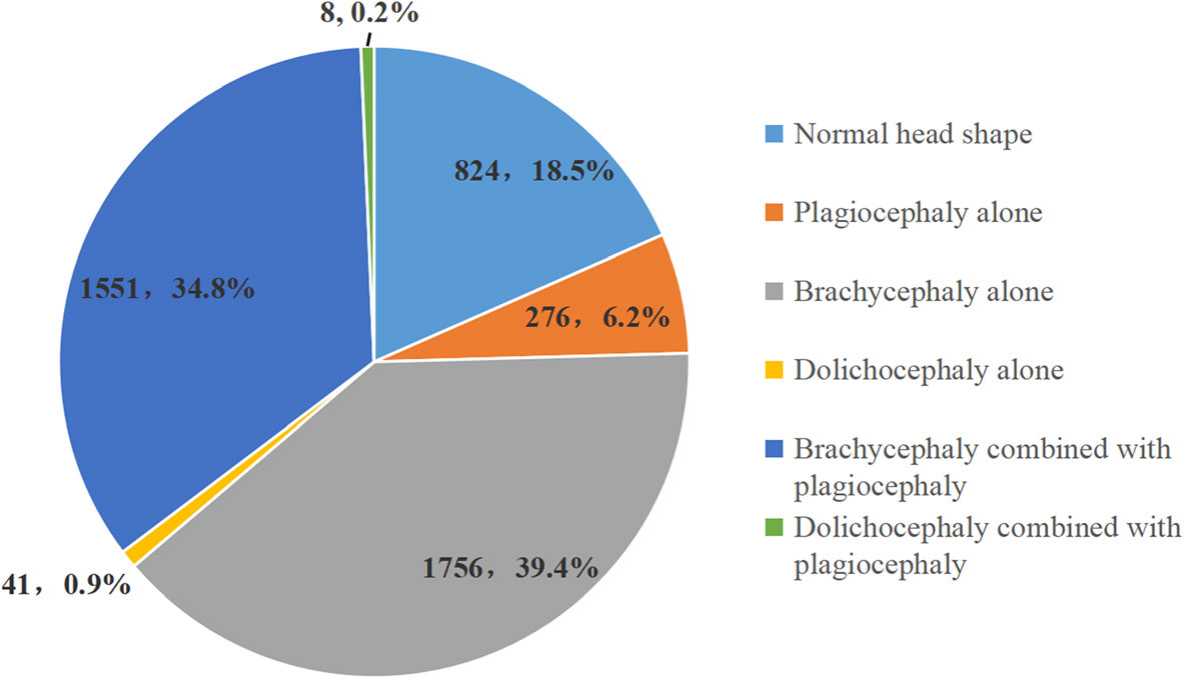
Distribution of cranial patterns in term infants (*n*, %)

According to the current international standards, among the 4456 term infants, 3632 cases (81.5%) of PHD and 824 cases of normal cranial type (18.5%) were detected. In the classification of abnormal skull type, the detection rate of brachycephaly alone was the highest (1756 cases, 39.4%) followed by brachycephaly combined with plagiocephaly (1551 cases, 34.8%) and plagiocephaly alone (276 cases, 6.2%), while dolichocephaly alone (41 cases, 0.9%) and dolichocephaly combined with plagiocephaly (8 cases, 0.2%) were relatively low (Fig. 2).

### Plagiocephaly in each age group

According to the current international standards, the total number of studied infants with plagiocephaly was 1835 (41.2%). The detection rate of plagiocephaly in the 0-1months group (35.9%) was significantly lower than that among the 2–3 (44.5%) and 4–5 months groups (43.9%). There were no significant differences between the 2–3 and 4–5 months groups. Regarding the severity of plagiocephaly, mild plagiocephaly was the highest in each age group, and the detection rate of mild, medium, and severe plagiocephaly in the 2–3 and 4–5 months groups were significantly higher than those in the 0–1 month group; however, there were no significant differences in the detection rate of each degree of plagiocephaly between the 2–3 and 4–5 months groups (Table 3).

**Table 3:** Plagiocephaly severity in term infants of different age groups

Group (age, months)	*n*	CVA (cm)	Plagiocephaly	Severity		
				Mild	Medium	Severe
0–1	1628	0.27 ± 0.25	584 (35.9%)	550 (33.8%)	24 (1.5%)	10 (0.6%)
2–3	1506	0.37 ± 0.29	670 (44.5%)^*^	614 (40.8%)^*^	32 (2.1%)^*^	24 (1.6%)^*^
4–5	1322	0.34 ± 0.29	581 (43.9%)^*^	517 (39.1%)^*^	35 (2.6%)^*^	29 (2.2%)^*^

Note: ^*^ significant difference compared to the 0–1 months group (*P* < 0.05)

*CVA* Cranial vault asymmetry

### Brachycephaly in each age group

According to the current international standards, the total number of studied infants with brachycephaly was 3307 (74.2%). The detection rate in the 0–1 month group (66.1%) was significantly lower than that in the 2–3 (82.0%) and 4–5 months groups (75.3%). There were no significant differences between the 2–3 and 4–5 months groups. Regarding the severity of brachycephaly, mild brachycephaly was the highest in each group. Medium and severe brachycephaly in the 2–3 and 4–5 months groups were significantly higher than those in the 0–1 months group, while the detection rate of mild brachycephaly was lower than that in the 0–1 months group. There were no significant differences between the 2–3 and the 4–5 months groups (Table 4).

**Table 4:** Brachycephaly severity in term infants of different age groups

Group (age, months)	*n*	CI (%)	Brachycephaly	Severity		
				Mild	Medium	Severe
0–1	1628	84.4 ± 5.4	1076 (66.1%)	827 (50.8%)	241 (14.8%)	8 (0.5%)
2–3	1506	88.0 ± 6.6	1235 (82.0%)^*^	671 (44.6%)^*^	514 (34.1%)^*^	50 (3.3%)^*^
4–5	1322	88.4 ± 6.0	996 (75.3%)^*^	566 (42.8%)^*^	390 (29.5%)^*^	40 (3.0%)^*^

Note: * significant difference compared to the 0–1 months group (*P* < 0.05)

*CI* Cephalic index

### Dolichocephaly in each age group

According to the current international standards, the total number of studied infants with dolichocephaly was 49 (1.1%). There were no significant differences in the detection rate of dolichocephaly among all age groups. Regarding the severity of dolichocephaly, mild dolichocephaly was dominant in all age groups, and no severe cases were detected (Table 5).

**Table 5:** Dolichocephaly severity in term infants of different age groups

Group (age, months)	*n*	Dolichocephaly	Severity		
			Mild	Medium	Severe
0–1	1628	16 (1.0%)	14 (0.9%)	2 (0.2%)	0
2–3	1506	19 (1.2%)	16 (1.1%)	3 (0.2%)	0
4–5	1322	14 (1.1%)	12 (0.9%)	2 (0.2%)	0

### Left and right plagiocephaly

**Fig. 3:**
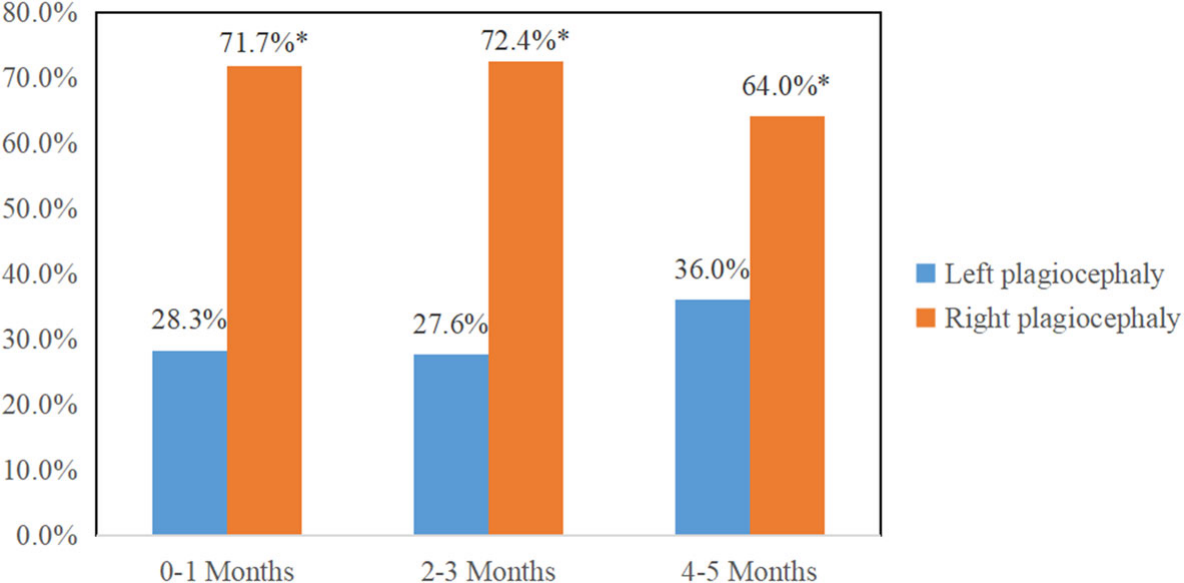
Left and right plagiocephaly in term infants in each age group *Statistically significant difference (*P* < 0.05)

Among the 1835 term infants diagnosed with plagiocephaly, right plagiocephaly (69.5%) was significantly higher than left plagiocephaly both overall and in each age group (Fig. 3).

### Percentile distribution of CVA and CI

The percentile method was used for analysis, and the percentile distribution of CVA and CI values grouped by age is shown in Table 6. The percentages CVA of 75th (P75)/90th (P90)/97th (P97) percentiles and CI values of 3rd (P3)/10th (P10)/25th (P25)/50th (P50)/P75/P90/P97 percentiles are 0.4/0.7/1.0 and 76.4/78.8/82.3/86.7/91.1/94.6/99.2%, respectively.

**Table 6:** Percentile distribution of CVA and CI in term infants

Percentile	0–1 months		2–3 months		4–5 months		0–5 months	
	CVA	CI	CVA	CI	CVA	CI	CVA	CI
3	0.0	74.8%	0.0	77.3%	0.0	78.6%	0.0	76.4%
10	0.1	77.4%	0.1	79.7%	0.1	80.6%	0.1	78.8%
25	0.1	80.1%	0.2	83.9%	0.1	83.8%	0.1	82.3%
50	0.2	84.7%	0.3	88.3%	0.2	87.8%	0.2	86.7%
75	0.4	88.3%	0.5	92.3%	0.5	92.7%	0.4	91.1%
90	0.5	91.7%	0.7	96.3%	0.7	96.8%	0.7	94.6%
97	0.9	93.8%	1.1	100.0%	1.0	101.5%	1.0	99.2%

*CVA* Cranial vault asymmetry, *CI* Cephalic index

## Discussion

Since the American Academy of Pediatrics advocated for “supine sleep” in the 1990s, the incidence of sudden infant death syndrome decreased significantly. However, the incidence of PHD increased significantly [5]. Since then, PHD has been widely studied. However, to the best of our knowledge, there are no detailed statistical and analytical reports on infant cranial types in China.

In our results, PHD incidence among 4456 term infants in Chongqing was 81.5% (Fig. 2) according to the international general diagnosis standards. Among them, the incidences of plagiocephaly (44.5%; Table 3) and brachycephaly (82.0%; Table 4) were the highest in the 2–3 months group, suggesting that the PHD incidence gradually increases up to 2–3 months after birth before gradually declining. This is because the infant’s head is not vertically stable for up to three months after birth, and caregivers usually place the infant in a supine position, where the occipital force is greater, leading to higher and lower incidences of brachycephaly and dolichocephaly, respectively (Table 5). In addition, at this stage, the infants’ ability to keep their head centered is poor. In cases where the head is inclined when supine, long term compression of the side of the skull results in plagiocephaly. At four months of age, their head control improves, the time spent outside the bed increases, uneven stress of the skull reduces, and, therefore, further aggravation of PHD decreases. Therefore, the first four months after birth is the key period for monitoring cranial shape, which should be measured monthly. Early detection of PHD and the corresponding correction are often effective in treating PHD.

In addition, this study found that the detection rate of right plagiocephaly in term infants of each age group was significantly higher than that of left (Fig. 3), which was consistent with the findings of Kluba et al. [15]. This may be because the apex of most fetal heads in the womb are located in the birth canal, with the left occipital side in front, such that the right occipital bone is pressed on the woman’s pelvis and the left forehead is in contact with the lumbosacral vertebrae. This is likely to continue after childbirth owing to sleeping posture because infants preferentially turn their heads to the right side to be comfortable, thus, aggravating the deformity on the right side [16].

This study also found that according to international general diagnostic standards, brachycephaly was frequent among the studied term infants. The rate of brachycephaly at the age of 2–3 months was 82.0%, which was much higher than that reported by Ballardini et al. [17]. However, the rate of plagiocephaly (44.5%) was similar to that reported by Mawji et al. (46.6%) [5] suggesting that the heads of infants in Chongqing are relatively flat. This relates to differences in parenting culture, customs, and esthetic preferences in different regions and nationalities. The flat head is in line with the esthetic preferences of Chinese parents. According to traditional Chinese parenting habits, the infant is mainly placed in the supine position after birth and, therefore, their head shape is relatively flat. In contrast, CVA, used to diagnose oblique head deformity, reflects the difference in stress conditions on the left and right sides of the head. As the ideal of bilateral skull symmetry is shared by Chinese and international parents, little difference was observed regarding this aspect. There are obvious differences between the basic data of cranial types of infants in this region and internationally. Therefore, it is inappropriate to apply the commonly used international standards to diagnose infants’ with PHD in this region.

At present, CVA ≥ 0.3 cm, CI ≥ 82%, and CI ≤ 76% indicate plagiocephaly, brachycephaly, and dolichocephaly [1, 7, 8], respectively. However, none of these suggestions were based on the “norm” of comprehensive statistical analysis, and there are fewer studies in Asian regions. Therefore, according to our findings, we considered the percentiles P25, P10, and P3 as the cutoff values for PHD, medium PHD, and severe PHD, respectively and put forward preliminary reference values for PHD diagnosis in infants younger than 6 months of age in Chongqing (Table 6). The diagnostic standards for brachycephaly and dolichocephaly are quite different from the international standards and are more suitable for the heads of Chinese infants and in line with Chinese parenting habits and esthetic preferences. It is noteworthy that deviation from CVA or CI values in infants aged 1–2 months is lesser than in those aged > 2 months. Nonetheless, we include all infants aged up to six months when we recommend the diagnostic standard, mainly because lower complexity makes it more convenient for primary health care institutions to diagnose children. In addition, we referred to the current international diagnostic standards, which did not distinguish the diagnostic criteria for different ages in months in detail. However, for the same reason, when a 0-1-month-old infant develops medium or severe PHD it suggests that the infant’s head deformity may be more serious and the risk higher; hence, full attention should be paid to correcting it in time.

In the assessment, diagnosis, and treatment of infant cranial measurement and PHD, repeated measurements are needed and, therefore, the accuracy and convenience of measurement methods are important issues for clinical workers to consider. This study adopted the manual measurement method performed by Wilbrand et al. [11], which requires simple equipment, little time or effort, and can be used repeatedly. After training, the measurement values of different research centers can reach consistency. Thus, it is an effective method that is suitable for use in primary health care institutions. However, in the process of using the bending foot gauge, there is a certain potential safety hazard when infants are crying or are uncooperative and special care should be provided.

The effectiveness of PHD correction is closely related to the growth rate of the skull [18]. The head grows rapidly before six months of age and skull hardness is low. The earlier PHD is detected, the better is the correction effect and the lower is the treatment cost. However, after six months, hardness of the skull increases, growth speed of the head circumference decreases, and the therapeutic effect decreases significantly [19, 20]. Therefore, early screening, diagnosis, and intervention should be performed. There are several limitations to our study. China is vast and has significant regional differences in environment and ethnicity; howere, our study only covers Chongqing area, it is unclear whether the acquisition of major motor milestones by infants of different ethnicities has a direct impact on the development of cranial type, still need further study. Another limitation is the long-term follow-up of cranial changes with the development, intervention methods, and their effects in term infants might be more informative.

## Conclusions

This study was the first to analyze large measurement data samples of the cranial patterns of term infants in mainland China, and we proposed preliminary local diagnostic reference standards according to the characteristics of the cranial patterns of Chinese infants. Our findings would help the prevention and treatment of PHD in infants from this region and other regions of China. Future research should collect the cranio-type data of infants from different regions and races in China to develop an in-depth understanding of PHD and identify influencing factors for the growth and development process of skulls to establish a unified Chinese standard.

## Data Availability

The dataset being analyzed/used during the current study is available from the corresponding author on reasonable request.

## Abbreviations

CI: Cephalic index
CVA: Cranial vault asymmetry
PHD: Positional head deformity
DA: Diagonal A
DB: Diagonal B
